# Sustained Improvement of Cognition, Mood and Plasma Markers Three Years After Metabolic Bariatric Surgery. The BARICO Study

**DOI:** 10.1007/s11695-025-08198-5

**Published:** 2025-08-28

**Authors:** Emma Custers, Debby Vreeken, Robert Kleemann, Roy P.C. Kessels, Esmee Tijman op Smeijers, Joachim Høg Mortensen, Martine C. Morrison, Eveline Gart, Maximilian Wiesmann, Eric J. Hazebroek, Amanda J. Kiliaan

**Affiliations:** 1 Department of Medical Imaging, Anatomy, Radboud Alzheimer Center, Radboud University Medical Center, Donders Institute for Brain, Cognition, and Behavior, Nijmegen, The Netherlands; 2 Department of Bariatric Surgery, Vitalys, Part of Rijnstate Hospital, Arnhem, The Netherlands; 3 Department of Metabolic Health Research, Netherlands Organisation for Applied Scientific Research (TNO), Leiden, The Netherlands; 4 Centre of Excellence for Korsakoff and Alcohol-related Cognitive Disorders, Vincent van Gogh Institute for Psychiatry, Venray, The Netherlands; 5 Donders Institute for Brain, Cognition and Behavior, Radboud University, Nijmegen, Netherlands; 6 Biomarkers & Research, Nordic Bioscience A/S, Herlev, Denmark; 7 Division of Human Nutrition and Health, Wageningen University, Wageningen, The Netherlands

**Keywords:** Metabolic bariatric surgery, Cognitive function, Obesity, Plasma biomarkers, Long-term outcomes

## Abstract

**Background:**

The primary aim of this study is to investigate the impact of MBS induced weight loss on cognition, and secondary investigate the impact of this weight loss on adipokines, inflammatory factors, vascular markers, mood and physical activity three years after MBS.

**Methods:**

This observational study assessed data from 107 patients with severe obesity (aged 35 to 55 years) from the BARICO (BAriatric surgery Rijnstate and Radboudumc neuroImaging and Cognition in Obesity) study, eligible for Roux-en-Y gastric bypass. Data were collected before, and at 6, 24 and 36 months after MBS. The primary outcome was long-term cognitive improvement, assessed using the 20% change index, which compares postoperative to preoperative test scores across cognitive domains. Mood and physical activity were assessed using the Beck Depression Inventory (BDI) and the Baecke questionnaire, respectively.

**Results:**

In total, 107 participants (mean [SD] age, 46.8 [5.6] years; 91 [85%] female) were included. Three years after MBS, global cognition was at least 20% higher in 38.6% (n = 39) of the participants. Compared to baseline, inflammatory factors, leptin, matrix metalloproteinase-9 (MMP-9), and apolipoprotein A1 (ApoA1) levels remained lower (median [IQR] C-reactive protein: 4.51 [2.96–8.35] vs 0.60 [0.30–1.30] µg/ml; p < 0.001; serum amyloid-alpha: 6.94 [4.80–15.16] vs 3.70 [2.30–6.00] µg/ml; p < 0.001; leptin: 64.6 [50.95–85.91] vs 18.95 [11.05–33.38] pg/ml; p < 0.001; MMP-9: 22.2 [18.2–31.3] vs 16.8 [13.2–23.1] ng/ml; p < 0.001; ApoA1: 535.47 ± 150.94 (SD) vs 261.33 ± 112.75 (SD) µg/ml; p < 0.001), whereas adiponectin (2.20 [1.70–2.75] vs 4.80 [3.50–7.00] µg/ml; p < 0.001) and angiopoietin-1 (ANGPT-1: 14.3 [10.4–22.55] vs 26.15 [21.2–33.9] ng/ml; p < 0.001) levels remained higher three years after MBS. Additionally, depressive symptoms remained low three years after MBS (median [IQR] BDI score: 9 [5.25–13] vs 4 [2–7]; p < 0.001), whereas physical activity returned to baseline.

**Conclusion and Relevance:**

Three years after MBS, weight loss remains associated with improved cognition and general health, evidenced by lower blood pressure, lower medication use, less systemic inflammation, lower leptin and higher adiponectin levels, and improved vascular markers.

**Supplementary Information:**

The online version contains supplementary material available at 10.1007/s11695-025-08198-5.

## Introduction

The prevalence of obesity continues to increase globally[1] and besides its impact on metabolic and cardiovascular health, midlife obesity can increase cognitive decline and dementia in later life[2, 3].

Several cognitive domains, including executive function, memory and verbal fluency, are often found to be decreased in obesity[4]. Studies have shown that metabolic bariatric surgery (MBS)-induced weight loss improved cognition at already three months, and persisted up to three years post-MBS [5, 6]. Nonetheless, mechanisms explaining these cognitive improvements remain largely unknown. Proposed processes involve changes in proinflammatory adipokines, as these may affect cerebral blood flow with concomitant neurodegeneration[7]. Additionally, improved mood and increased physical activity (PA) have been observed up to 4 years after MBS[8], and both of which are positively associated with improvements in cognition[9, 10]. In two sub-studies of the BARICO (Bariatric Surgery Rijnstate and Radboudumc Neuroimaging and Cognition in Obesity) study, it was found that cognitive improvement post-surgery was indeed associated with lower systemic inflammation, lower leptin levels, higher adiponectin levels, improved mood and PA 6 and 24 months post-surgery[11, 12].

The primary objective of this study is to investigate the long-term effects of metabolic bariatric surgery (MBS)-induced weight loss on cognitive function. The secondary aim is to investigate the longitudinal changes of general health, anthropometric measurements, plasma biomarkers, PA and mood en examine associations between these measurements and cognitive changes and the differences of the changes in anthropometric measurements, plasma biomarkers, PA and mood between patients with and without cognitive improvement. This study will improve our understanding of long-term consequences of MBS-induced weight loss on cognitive function as well as underlying mechanisms. Eventually this may improve strategies for obesity and cognitive impairment.

## Methods

### Study Population

**Fig. 1:**
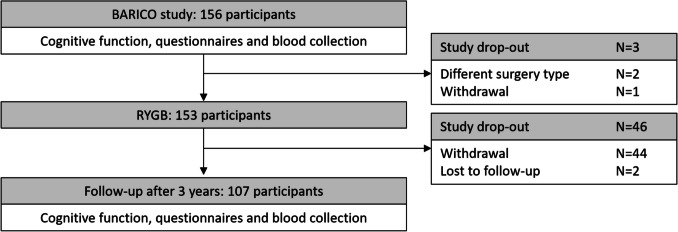
Flow chart of the study. In total 156 participants were included and underwent baseline measurements. Of those, 107 completed the 3 years follow-up measurements. Abbreviations: RYFB: Roux-en-Y Gastric Bypass

Data were obtained from the BARICO study. Participants between 35 and 55 years old, eligible for Roux-en-Y gastric bypass, were recruited at Rijnstate Hospital (Arnhem, the Netherlands, between September 2018 and December 2020). Exclusion criteria were: pregnancy, neurologic or severe psychiatric illness, and treatment with antibiotics, pro- or prebiotics, 3 months before, and at any timepoint during the study. Neuropsychological tests were performed before (baseline), and at 6, 24 and 36 months post-MBS. At each timepoint blood samples and anthropometric data were collected. Only participants who completed all measurements 36 months after surgery were included (Fig. 1).

### Standard Protocol Approvals, Registrations, and Patient Consents

The medical ethics committee CMO region Arnhem – Nijmegen (NL63493.091.17) and the local institutional ethics committee approved this study. The study was performed according to the Declaration of Helsinki ‘Ethical Principles of Medical Research Involving Human Subjects’ and in agreement with the guidelines for Good Clinical Practice (CPMP/ICH/135/95). All participants signed written informed consent. The study was registered in the Netherlands Trial Registry (https://onderzoekmetmensen.nl/nl/trial/55792).

### Medical Examination

Anthropometric measurements included body weight, body mass index (BMI), waist circumference (WC), and percentage total body weight loss (%TBWL). BMI was determined as weight divided by height in meters squared. %TBWL was defined as: $\frac{starting weight-current weight}{starting weight}x100$. Systolic and diastolic blood pressure were measured in sitting position.

### Blood Samples

Fasting blood samples were collected at all timepoints and stored at −80 °C. Plasma leptin, adiponectin, C-reactive protein (CRP), growth differentiation factor-15 (GDF-15), apolipoprotein-A1 (ApoA1), matrix metalloproteinase-9 (MMP-9), angiopoietin-1 (ANGPT-1), serum amyloid A (SAA), plasminogen activator inhibitor-1 (PAI-1) and brain derived neurotrophic factor (BDNF) were determined with human enzyme-linked immunosorbent assays (ELISAs). Tumor necrosis factor alpha (TNF-α), interleukin-1β (IL-1β) and IL-6 were determined by electrochemiluminescence using established multiplex panels (Quanterix, Billerica, USA; MesoScale Discovery, Rockville, Maryland, USA) according to manufacturer’s protocols. Details regarding ELISA assays and electrochemiluminescence are listed in eTable 1.

### Cognitive Outcomes

General cognition was assessed using the Montreal Cognitive Assessment (MoCA)[13], working memory by combining results of the 3 trials (forward, backward and sorting) of the Digit Span test (Wechsler Adult Intelligence Scale-Fourth Edition)[14] and the immediate and delayed Story Recall subtest from the Rivermead Behavioral Memory Test was used to assess episodic memory[15]. Verbal fluency was assessed with the Controlled Oral Word Association test (COWAT) and ability to shift attention with the Flexibility subtest from the computerized Test of Attentional performance (TAP, version 2.3.1)[16]. To prevent material-specific practice effects, parallel versions of the MoCA, story recall and COWAT were used for each time point. The MoCA score (maximum = 30) reflects the total number of correct responses[13]. The Digit Span score was derived by summing the number of correctly recalled sequences across the forward, backward, and sorting trials (Digit Span Total = Forward + Backward + Sorting)[14]. The Story Recall score represents the combined number of correctly recalled items from both the immediate and delayed recall trials (Story Recall Total = Immediate Recall + Delayed Recall)[15]. The COWAT score corresponds to the total number of correct, non-repeated words generated across three phonemic trials (COWAT Total = Sum of correct words across three phonemic trials)[17]. The TAP score was extracted from the flexibility index, which is automatically computed by the TAP software (version 2.3.1) based on reaction time and accuracy during task switching[16]. A compound Z-score of global cognition was calculated with the mean of the z-scores of each subtest, ranging from −1.57 to 1.67, with higher scores indicating better cognitive performance. The z-scores at 6, 24 and 36 months post-surgery were based on the mean (SD) of each test score at baseline. Education level was determined using the Verhage score[18] based on the Dutch educational system, comparable with the International Standard Classification of Education[19].

To examine associations of MBS with cognition, we calculated the 20% change index[20] 36 months post-surgery due to the absence of a control group and we used standardized alternate versions of the cognitive tests to reduce potential practice effects. This 20% change index indicates cognitive improvements if a participant’s postoperative test score is 20% higher compared to their preoperative test score, defined as: $\frac{5( \text{X}2-\text{X}1)}{\text{X}1}$. Here, X_2_ is the participant’s postoperative score and X_1_ the preoperative score. Calculations were performed for each cognitive domain and compound Z-score. An index ≥ 1.00 indicates clinically significant cognitive improvement[20]. While this approach provides a practical representation of cognitive change, we acknowledge that more rigorous methods—such as the Reliable Change Index (RCI) or inclusion of a control group—are generally preferred. However to calculate the RCI, you need reference values from repeated measurements and the correlations between all tests in control or normative groups. The BARICO study does not have its own control group and that’s why we ultimately opted for the 20% change index which given the study design, is the best achievable option. Due to the observational nature of the study, no causal conclusions can be drawn. Future randomized controlled trials are necessary to validate these findings and clarify the underlying mechanisms.

Global cognitive function was assessed using a composite score[21], calculated as the mean of the z-scores from the natural log-transformed MoCA score, the flexibility subtest from the Tests of Attentional Performance, the combined score from three Digit Span trials (forward, backward, and sorting), the total verbal fluency score, and the sum of the immediate and delayed recall scores from the story recall test.

### Questionnaires

Participants filled out standardized online questionnaires at each timepoint. Depressive symptoms, later referred to as mood, were assessed via the 21-item self-reported Beck Depression Inventory (BDI) [22]. The BDI determines presence of depressive symptoms over the past 2 weeks. Questions were rated from 0 (no symptom) to 3 (severe symptom). Minimal depression was indicated by a total score of 0 to 9, mild by 10 to 20, moderate by 21 to 30, and severe depression by ≥ 31. The total BDI score was calculated by summing the item scores (BDI Total = Sum of 21 item scores)[22]. PA was assessed using the Baecke questionnaire[23], including 16 questions with a 5-point Likert scale about time spent on different activities. Index scores for work, sport and leisure were combined into a total PA score (PA Total = Work Index + Sport Index + Leisure Index). The total score ranges from 3 to 15, with higher scores indicating a higher level of PA.

### Statistical Analysis

The sample size was based on the BARICO study protocol[24], which estimated that 150 participants would provide 90% power to detect a standardized effect size of ≥ 0.3 on neuropsychological outcomes, including the Digit Span task. The current analysis focuses on long-term cognitive improvement as the primary outcome, while comparisons between cognitive improvers and non-improvers were exploratory. Statistical analyses were performed using SPSS Statistics version 29 (IBM). Continuous variables were checked for normality, and if not met, they were presented as median (IQR). Linear mixed models with Bonferroni correction (correcting for multiple comparisons) and Cochran test assessed changes over time for continuous and categorical data. We controlled for age, sex, education and preoperative BMI. These covariates were selected based on their established relevance to both cognitive and metabolic outcomes, and to align with our primary aim—to investigate the long-term effects of MBS-induced weight loss on cognitive function—and our secondary aim to explore associations between changes in general health, plasma biomarkers, mood, and physical activity with cognitive outcomes. To preserve statistical power and avoid overfitting in our cohort, we limited the number of covariates to those most directly relevant to our hypotheses.

Baseline differences in age, sex, and education between cognitive improvers and non-improvers (based on the 20% change index) were assessed using independent t-tests and chi-square tests (eTable 2), while longitudinal group differences were analyzed using linear mixed models (Table 3). Linear mixed models were used to determine differences between groups regarding %TBWL, BMI, WC, circulating plasma markers, depressive symptoms and PA over time within- and between groups. Lastly, we investigated if changes in cognition were associated with changes in %TBWL, BMI, WC, circulating plasma makers, depressive symptoms and PA using bivariate correlations. Given the exploratory nature of these analyses and the number of comparisons, we interpreted the results with caution and emphasized associations that were consistent across cognitive domains and aligned with existing literature. To address the absence of a control group, we applied the 20% change index to detect meaningful within-subject cognitive improvement. This approach is suitable for observational designs and allowed us to quantify individual-level change over time. As sensitivity analysis, we repeated the analyses in women only. Missing variables are presented in eTable 3. P-values were 2-sided, and p < 0.05 was considered statistically significant. Data were analysed from July to October 2024.

## Results

### Descriptive Statistics

In total, 107 participants (mean [SD] age, 46.8 [5.6] years; 91 [85%] female) were included (Table 1). Mean body weight, BMI, WC, blood pressure and medication use were still significantly lower three years post-surgery. Compared to baseline, the MoCA score increased significantly three years post-surgery.

**Table 1:** Patient characteristics (*n* = 107)

	**Mean ± SD**														
	**All participants (n = 107)**					**Female (n = 91)**					**Male (n = 16)**				
	**Baseline**	**6 mo**	**2 y**	**3 y**	**P**	**Baseline**	**6 mo**	**2 y**	**3 y**	**p**	**Baseline**	**6 mo**	**2 y**	**3 y**	**p**
**Age, y**	46.8 ± 5.6	n/a	n/a	n/a	n/a	46.46 ± 5.52	n/a	n/a	n/a	n/a	48.76 ± 5.51	n/a	n/a	n/a	n/a
**Body weight, kg**	122.48 ± 16.06	89.32 ± 12.98	80.64 ± 13.74	83.28 ± 14.34	< 0.001	119.16 ± 14.0	87.15 ± 11.94	77.62 ± 11.97	80.52 ± 13.06	< 0.001	141.36 ± 14.17	101.68 ± 12.03	97.82 ± 10.20	98.96 ± 11.09	< 0.001
**BMI, kg/m**^**2**^	41.82 ± 4.11	30.42 ± 3.81	27.44 ± 3.87	28.34 ± 4.12	< 0.001	41.70 ± 4.21	30.44 ± 3.93	27.12 ± 3.94	28.12 ± 4.29	< 0.001	42.34 ± 3.43	30.38 ± 2.94	29.26 ± 2.76	29.58 ± 2.72	< 0.001
**WC, cm**^**a**^	124.29 ± 11.62	100.13 ± 11.65	95.18 ± 10.53	97.32 ± 12.37	< 0.001	122.07 ± 10.93	99.45 ± 11.58	94.92 ± 11.66	94.67 ± 13.21	< 0.001	136.67 ± 5.90	108.42 ± 10.60	103.25 ± 8.0	105.32 ± 9.35	< 0.001
**TBWL, %**	n/a	27.05 ± 5.04	34.14 ± 7.10	31.98 ± 7.65	< 0.001	n/a	26.86 ± 5.21	34.75 ± 7.26	32.35 ± 7.94	< 0.001	n/a	28.13 ± 3.85	30.66 ± 5.03	29.89 ± 5.43	0.020
**Level of education, n (%)**^**b**^															
Low	6 (5.6)	n/a	n/a	n/a	n/a	6 (6.6)	n/a	n/a	n/a	n/a	0 (0)	n/a	n/a	n/a	n/a
Middle	62 (57.9)	n/a	n/a	n/a	n/a	52 (57.1)	n/a	n/a	n/a	n/a	10 (62.5)	n/a	n/a	n/a	n/a
High	39 (36.4)	n/a	n/a	n/a	n/a	33 (36.3)	n/a	n/a	n/a	n/a	6 (37.5)	n/a	n/a	n/a	n/a
**Use of medication, n (%)**^**c**^															
Oral antidiabetics	9 (8.4)	5 (4.7)	2 (1.9)	5 (4.7)	0.055	7 (7.7)	4 (4.4)	2 (2.2)	5 (5.6)	0.181	2 (12.5)	1 (6.3)	0 (0)	0 (0)	0.194
Insulin therapy	7 (6.5)	3 (2.8)	3 (2.8)	2 (1.9)	0.007	7 (7.7)	3 (3.3)	3 (3.3)	2 (2.2)	0.007	0 (0)	0 (0)	0 (0)	0 (0)	n/a
Blood pressure lowering agents	37 (34.6)	22 (20.6)	16 (15)	17 (16)	< 0.001	29 (31.9)	16 (17.6)	10 (11.0)	12 (13.3)	< 0.001	8 (50.0)	6 (37.5)	6 (37.5)	5 (31.3)	0.096
Lipid lowering agents	14 (13.1)	9 (8.4)	9 (8.4)	10 (9.4)	0.026	10 (11.0)	6 (6.6)	6 (6.6)	7 (7.8)	0.079	4 (25.0)	3 (18.8)	3 (18.8)	3 (18.8)	0.392
Antidepressants	10 (9.3)	8 (7.5)	9 (8.4)	6 (5.7)	0.241	10 (11.0)	8 (8.8)	9 (9.9)	5 (5.6)	0.054	0 (0)	0 (0)	0 (0)	1 (6.3)	0.392
**Blood pressure, mm Hg**^**d**^															
Systolic	138.44 ± 15.21	126.72 ± 16.21	128.60 ± 16.97	128.07 ± 16.44	< 0.001	136.51 ± 15.83	125.99 ± 16.73	127.07 ± 16.28	125.81 ± 19.59	< 0.001	148.56 ± 15.15	129.92 ± 17.90	139.94 ± 22.92	135.86 ± 19.77	< 0.001
Diastolic	85.5 ± 7.29	80.19 ± 9.94	80.07 ± 10.58	78.16 ± 7.69	< 0.001	84.51 ± 8.01	80.40 ± 9.91	79.18 ± 10.50	78.24 ± 8.37	< 0.001	91.63 ± 11.72	82.23 ± 11.70	84.94 ± 13.16	82.64 ± 6.85	0.008
**MoCA score, median (IQR)**	27 (26–29)	27 (25–28)	27 (25–28)	28 (26–29)	0.006	28 (26–29)	27 (26–28)	27 (25–28)	28 (27–29)	< 0.001	26 (24–7)	26 (25–27.5)	26 (24.3–26.8)	24.5 (23.5–27)	0.356

Linear mixed models and Cochran test were conducted to examine changes in characteristics over time. ^a^ Complete data on all timepoints were available for 75 participants. ^b^ A Verhage score of ≤ 4 is defined as low level of education; 5, middle level; and 6 or 7, high level[18]. ^c^ Complete data on all timepoints were available for 106 participants. ^d^ Complete data on all timepoints were available for 73 participants. Abbreviations: *BMI* body mass index, *WC* waist circumference, *TBWL* total body weight loss, *MoCA* Montreal cognitive assessment, *n/a* not applicable, *mo* months, *y* years, *p p*-value

### Cognition, Mood and Physical Activity

**Fig. 2:**
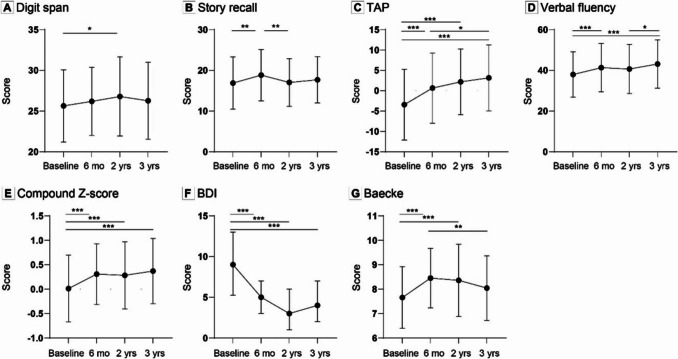
Changes in cognitive outcomes, physical activity and depression symptoms among bariatric surgery patients (*n* = 107). Repeated measures analyses of variance were conducted to examine changes over time. Significant changes over time are indicated by stars. (**a**) Change in digit span (sum of forward, backward and sorting) over time. (**b**) change of story recall (sum of immediate and delayed recall) over time. (**c**) Change in TAP flexibility index score over time, complete data on all timepoints were available for 101 participants. (**d**) Change in controlled oral word association test over time. (**e**) Change in general cognition (based on compound Z-score) over time, complete data on all timepoints were available for 101 participants. (**f**) Changes in depressive symptoms over time, complete data on all timepoints were available for 92 participants. (**g**) Changes in physical activity over time, complete data on all timepoints were available for 58 participants. Values are presented as mean(SD), only values of BDI are presented as median(IQR). * p < 0.05, ** p < 0.01, ***p < 0.001. Abbreviations: TAP = test of attentional performance, BDI = beck depression inventory

Cognitive test scores were higher 3 years post-MBS compared to baseline (Table 2, Fig. 2). Working memory (digit span) (p = 0.047), episodic memory (story recall) (p < 0.001), verbal fluency (COWAT) (< 0.001), ability to shift attention (TAP test) (p < 0.001) and global cognitive performance (compound Z-score) (p < 0.001) significantly improved over time.

**Table 2:** Changes in cognitive outcomes, depression symptoms and physical activity among metabolic bariatric surgery patients (*n* = 107)

	**Mean ± SD**					
	**Baseline**	**6 months**	**2 years**	**3 years**	**p-value**	** ≥ 20%, n (%)**
**Cognition**						
Digit Span (sum of forward, backward, and sorting)^a^	25.64 ± 4.43	26.19 ± 4.17	26.79 ± 4.86	26.25 ± 4.72	0.047	11 (10.3)
Story Recall (sum of immediate and delayed recall)	16.87 ± 6.43	18.82 ± 6.30	17.01 ± 5.86	17.67 ± 5.65	< 0.001	38 (35.5)
COWAT	37.93 ± 11.12	41.29 ± 11.94	40.61 ± 12.06	43.06 ± 11.8	< 0.001	40 (37.4)
TAP flexibility index score^b^	−3.27 ± 8.93	0.94 ± 8.57	2.13 ± 8.40	3.47 ± 8.20	< 0.001	59 (58.4)
Compound Z-score^b^	0.026 ± 0.68	0.32 ± 0.63	0.33 ± 0.66	0.42 ± 0.65	< 0.001	39 (38.6)^c^
**BDI**^**a**^						
Median (IQR)	9 (5.25–13)	5 (3–7)	3 (1–6)	4 (2–7)	< 0.001	n/a
Minimal, n (%)	55 (52.9)	91 (87.5)	91 (89.2)	84 (85.7)	n/a	n/a
Mild, n (%)	45 (43.3)	13 (12.5)	9 (8.8)	11 (11.2)	n/a	n/a
Moderate, n (%)	4 (3.8)	0 (0)	2 (2.0)	2 (2.0)	n/a	n/a
Severe, n (%)	0 (0)	0 (0)	0 (0)	1 (1.0)	n/a	n/a
**Baecke**^**d**^	7.55 ± 1.27	8.25 ± 1.22	8.05 ± 1.40	7.83 ± 1.12	< 0.001	n/a

Linear mixed models were conducted to examine changes in characteristics over time. ^a^ Corrected for sex. ^b^ Complete data on all timepoints were available for 92 participants. ^c^ Complete data on baseline and 3 years after surgery were available for 101 participants. ^d^ Complete data on all timepoints were available for 58 participants. Post-hoc comparisons are depicted in eFigure 2. Abbreviations: *COWAT* controlled oral word association test, *TAP* test of attentional performance, *BDI* Beck Depression Inventory, *n/a* not applicable

Based on the 20% change index, 11 participants (10.3%) showed improvement in working memory, 38 participants (35.5%) showed improvement in episodic memory, 40 participants (37.4%) showed improvement in verbal fluency and 59 participants (58.4%) showed improvement in ability to shift attention three years post-surgery compared to baseline. Finally, 39 participants (38.6%) showed improvement in global cognition three years post-surgery. According to the BDI questionnaire, depressive symptoms were lower six months post-surgery (p < 0.001) and remained low three years post-MBS. At baseline 45 participants (43.3%) had mild depressive symptoms, 4 participants (3.8%) showed moderate depressive symptoms, and none of the participants suffered from severe depressive symptoms. Three years post-surgery, only 11 (11.2%), 2 (2.0%) and 1 (1.0%) participants showed mild, moderate and severe depressive symptoms respectively. Detailed analysis revealed significantly higher PA six months (p < 0.001) and two years after MBS (p < 0.001), but returned to baseline three years post-surgery (Fig. 2).

### Plasma Markers

**Fig. 3:**
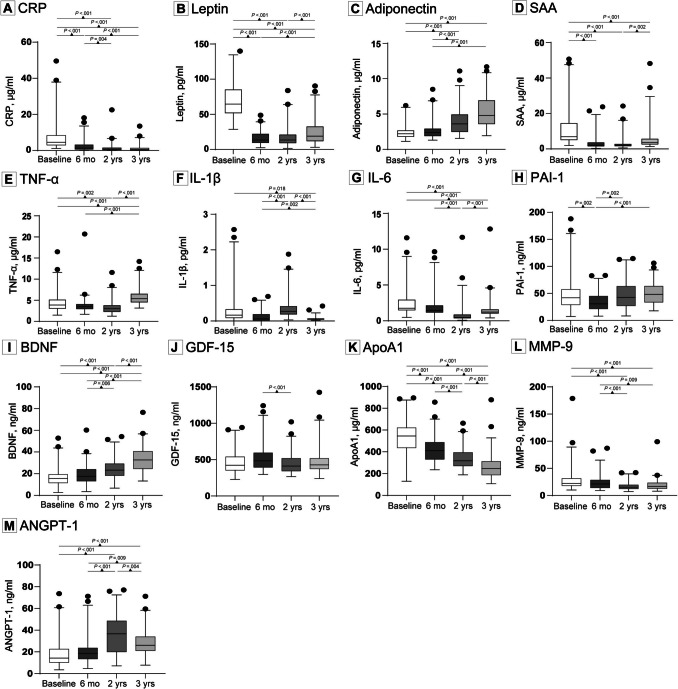
Plasma concentrations of adipokines and cytokines among patients who underwent bariatric surgery. Linear mixed models were conducted to assess changes in circulating factors over time. Significant changes over time are indicated by p-values. Complete data for all parameters on all timepoints were available for 85 participants, except for SAA (83 participants) and IL-1β, GDF-15 and ANGPT-1 (84 participants). For illustrative purposes one extreme high SAA value (111.49 µg/ml) at baseline, and one SAA value (88.82 µg/ml) at 6 months were not shown. For IL-1β, one extreme high value (7.59 pg/ml) at baseline and two extreme high values (3.26 pg/ml, 5.12 pg/ml) at 2 years were not included in the graph. Similarly, for GDF-15 on extreme high value (2269.20 ng/ml) at baseline was not shown. Individual values for every plasma marker are presented in eTable 4. Abbreviations: CRP = C-reactive protein, SAA = serum amyloid alpha, TNF-α = tumor necrosis factor-alpha, IL = interleukin, PAI-1 = plasminogen activator inhibitor-1, BDNF = brain derived neurotrophic factor, GDF-15 = growth differentiation factor-15, ApoA1 = apolipoprotein A1, MMP-9 = matrix metalloproteinase-9, ANGPT-1 = angiopoietin-1, mo = months, yrs = years

Changes in markers are depicted in Fig. 3 and eTable 4. CRP levels were significantly lower six months post-surgery (p < 0.001) and continued to decrease two (p = 0.004) and three year post-surgery (p < 0.001). Leptin and SAA levels were significantly lower six months post-surgery (p < 0.001, p < 0.001), remaining low at two- and three-year post-surgery (p < 0.001, p < 0.001). Adiponectin continued to increase three year post-surgery (< 0.001). TNF-α levels were significantly lower two years (p = 0.002), but increased at three years post-surgery (p < 0.001) with levels being higher than baseline (p < 0.001). Compared to the six months timepoint, IL-1β levels increased at two years (p < 0.001), but decreased at three years after surgery (p < 0.001), reaching significantly lower levels than baseline (p = 0.018). IL-6 was significantly reduced at two year (p < 0.001) but increased three year post-surgery (p < 0.001), yet remaining significantly lower than baseline (p < 0.001). PAI-1 was significantly reduced six months post-surgery (p = 0.002), and returned to baseline level in three years post-surgery. BDNF increased over time and was significantly higher two and three year post- surgery (p < 0.001). By contrast, GDF-15 levels were lower at two years compared to six months after MBS (p < 0.001) but no differences were found with baseline values at all time points post-surgery. ApoA1 decreased over time and was significantly lower six months (p < 0.001), two year (p < 0.001) and three year post-surgery (p < 0.001). MMP-9 was significantly lower two year (p < 0.001) and three year post-surgery (p < 0.001). ANGPT-1 levels were higher two years post-surgery (p < 0.001), and decreased three years post-surgery (p = 0.004), with its levels being still significantly higher than baseline (p < 0.001).

### Cognitive Improvers Versus Cognitive Non-Improvers

In total, 39 participants (38.6%) were identified as cognitive improvers and 62 (61.4%) were identified as non-improvers (n = 62, eTable 2). Baseline differences in demographic and cognitive characteristics between cognitive improvers and non-improvers are presented in eTable 2, while longitudinal changes over time between groups were analyzed using linear mixed models and are reported in Table 3. Most parameters showed similar effects over time between cognitive improvers and non-improvers. However, characteristic for cognitive improvers was the more pronounced reduction of median [IQR] leptin levels compared with non-improvers (p = 0.035) (leptin: cognitive improvers from 76.20 [61.80–91.65] pg/ml to 25.10 [13.20–40.90] pg/ml; p < 0.001, non-improvers from 59.50 [47.90–77.30] pg/ml to 18.45 [10.30–27.30] pg/ml; p < 0.001). Cognitive improvers also showed lower mean [SD] GDF-15 levels at baseline and three year post-surgery compared with cognitive non-improvers (p = 0.070; trend) (GDF-15: cognitive improvers from 418.43 [129.66] ng/ml to 436.69 [141.09] ng/ml; p = 0.516, non-improvers from 486.82 [168.16] ng/ml to 463.50 [191.24] ng/ml; p = 0.265). Regarding baseline cognition scores, cognitive improvers showed higher scores for digit span, COWAT, TAP and Compound Z-score compared to cognitive non-improvers (eTable 2). Sensitivity analysis showed similar results (eTable 5).

**Table 3:** Differences in anthropometric measures, plasma levels, mood and physical activity between cognitive improvers and non-improvers 3 years after metabolic bariatric surgery

	**Within groups**						**Between groups**
	**Improvers (n = 39)**			**Non-improvers (n = 62)**			**Linear mixed models**
	**Baseline**	**3 years**	**p**	**Baseline**	**3 years**	**p**	**p-value**
**Anthropometric measurements**							
TBWL (%), mean ± SD^a^		32.92 ± 7.79	n/a		31.17 ± 7.50	n/a	0.263
BMI (kg/m^2^), mean ± SD	42.09 ± 3.38	28.11 ± 4.10	< 0.001	41.58 ± 4.48	28.56 ± 4.09	< 0.001	0.964
WC (cm), mean ± SD^b^	121.87 ± 10.24	97.58 ± 14.05	< 0.001	125.95 ± 11.76	96.65 ± 12.46	< 0.001	0.871
**Blood pressure, mean ± SD**^**c**^							
Systolic (mm HG)	137.31 ± 16.26	122.19 ± 23.22	< 0.001	139.05 ± 16.22	130.02 ± 17.60	< 0.001	0.095
Diastolic (mm HG)	84.64 ± 6.84	77.86 ± 7.24	< 0.001	86.48 ± 9.53	79.11 ± 9.16	< 0.001	0.213
**Plasma levels, median (IQR)**							
CRP (µg/ml)	4.94 (2.96–10.4)	0.60 (0.40–1.40)	< 0.001	4.43 (3.08–8.49)	0.60 (0.30–1.30)	< 0.001	0.361
Leptin (pg/ml)^b^	76.20 (61.80—91.65)	25.10 (13.20—40.90)	< 0.001	59.50 (47.90—77.30)	18.45 (10.30—27.20)	< 0.001	0.035
Adiponectin (µg/ml)^b^	2.40 (1.95—3.05)	4.50 (3.90—6.30)	< 0.001	2.20 (1.70—2.80)	4.80 (3.70—7.30)	< 0.001	0.889
SAA (µg/ml)^d^	7.58 (5.29—16.63)	3.90 (2.40—8.10)	0.010	6.81 (4.18—12.30)	3.45 (2.20—5.60)	< 0.001	0.341
TNF-α (pg/ml)^b^	4.47 (2.81—5.27)	5.60 (4.16—6.50)	< 0.001	3.87 (3.04—5.29)	5.41 (4.69—6.70)	0.003	0.157
IL-1β (pg/ml)^e^	0.18 (0.05—0.35)	0.06 (0.04—0.09)	0.045	0.17 (0.08—0.38)	0.05 (0.04—0.07)	< 0.001	0.304
IL-6 (pg/ml)^b^	2.04 (1.40—3.09)	1.22 (0.87—1.55)	0.001	1.74 (1.39—3.01)	1.37 (0.96—1.75)	0.043	0.612
PAI-1 (ng/ml)	42.7 (33.8–55.3)	55.7(36.7–65.0)	0.651	41.9 (26.7–60.5)	47.0 (32.0–59.8)	0.959	0.258
BDNF (ng/ml)	17.0 (12.6–23.8)	34.0 (25.7–40.9)	< 0.001	15.6 (11.5–18.8)	32.5 (23.3–40.7)	< 0.001	0.080
GDF-15, mean ± SD (ng/ml)^e^	418.43 ± 129.66	436.69 ± 141.09	0.516	486.82 ± 168.16	463.50 ± 191.24	0.265	0.070
ApoA1, mean ± SD (µg/ml)^b^	511.26 ± 147.97	245.62 ± 86.79	< 0.001	535.41 ± 154.43	255.32 ± 101.43	< 0.001	0.774
MMP-9 (ng/ml)^b^	21.7 (14.9–29.8)	16.7(11.3–21.7)	0.033	23.35 (18.5–30.7)	17.05 (13.4–23.7)	< 0.001	0.713
ANGPT-1 (ng/ml)^b^	15.1 (10.2–25.5)	28.6 (21.3–35.9)	< 0.001	14.45 (11–21.8)	26.55 (21.3–35.9)	< 0.001	0.309
**BDI, median (IQR)**^**c**^	9 (6–13)	4.5 (1–8.25)	0.022	9.5 (4–14)	4 (2–6)	< 0.001	0.337
**Baecke, mean ± SD**^**f**^	7.47 ± 1.26	7.77 ± 1.21	0.086	7.72 ± 1.20	8.07 ± 1.33	0.153	0.075

^a^p-value based on an one-way ANOVA between improvers and non-improvers. ^b^ Complete data on both timepoints were available for 90 participants. ^c^ Complete data on both timepoints were available for 92 participants. ^d^ Complete data on both timepoints were available for 88 participants. ^e^ Complete data on both timepoints were available for 89 participants. ^e^ Complete data on both timepoints were available for 74 participants. Abbreviations: *TBWL* total body weight loss, *BMI* body mass index, *WC* waist circumference, *CRP* C-reactive protein, *SAA* serum amyloid alpha, *TNF-α* Tumor necrosis factor alpha, *IL* interleukin, *PAI-1* plasminogen activator inhibitor-1, *BDNF* brain derived neurotrophic factor, *GDF-15* growth differentiation factor-15, *ApoA1* apolipoprotein A1, *MMP-9* matrix metalloproteinase-9, *ANGPT-1* Angiopoietin-1, *BDI* Beck Depression Inventory

Correlation coefficients between the changes in cognition and changes in anthropometrics, plasma markers, mood and PA are shown in eTable 6. A greater increase in adiponectin three years post-surgery was negatively associated with global cognition (r = −0.226; p = 0.037). A greater increase in BDI scores was positively associated with episodic memory (r = 0.216; p = 0.035) and negatively associated with verbal fluency post-surgery (r = −0.205; p = 0.045). Greater increase in ability to shift attention was positively associated with BDNF (r = 0.237; p = 0.030) and negatively associated with PAI-1 (r = −0.269; p = 0.013) post-surgery. Greater decrease in GDF-15 was positively associated with global cognition post-surgery (r = 0.241; p = 0.028). Greater decrease in ApoA1 was associated with episodic memory (r = 0.239; p = 0.023) and global cognition post-surgery (r = 0.326, p = 0.002). Greater increase in ANGPT-1 was positively associated with ability to shift attention post-surgery (r = 0.248; p = 0.022). Correlation analyses in women only showed similar results (eTable 7).

## Discussion

We demonstrated that three years post-MBS improvements in general health, indicated by lower body weight, BMI, WC, blood pressure, medication use, systemic inflammation and leptin levels, higher adiponectin levels and improved mood are still present. Moreover, we observed lower MMP-9 and higher BDNF and ANGPT-1 levels at three years post-surgery. Ability to shift attention and verbal fluency, domains of executive function, continued to increase three years post-surgery, and based on the 20% change index, 38.6% of the participants showed significant cognitive improvement. These improvements in general health, mood and circulating inflammatory and vascular markers, might be responsible for cognitive improvement post-surgery.

In our cohort, the ability to shift attention and verbal fluency continued to increase three years post-MBS, while digit span and story recall performance returned to baseline values. This suggests that obesity-associated cognitive impairment is partly reversible and sustained during MBS-induced weight loss. Furthermore, these results indicate that on the long term, MBS is most beneficial for executive functioning, which is in line with previous studies demonstrating impaired executive functioning in patients with obesity[25] and improved executive functioning after MBS[6].

We observed cognitive improvement in 38.6% of the participants. This percentage of cognitive improvers is comparable to the six month[11] and two year timepoints[12] of the BARICO study.

With regard to their plasma biomarkers, cognitive improvers did not differ much from cognitive non-improvers and mainly showed a more pronounced reduction of leptin levels post-surgery. Leptin is important for hippocampal synaptic plasticity and memory[26]. However, obesity is associated with leptin resistance and thereby may induce cognitive decline. Restoring leptin signalling may constitute a valid approach to restore synaptic function and cognition[26], potentially explaining the greater decrease in leptin in the cognitive improvers. Of note, cognitive-improvers showed higher baseline leptin levels than non-improvers.

At both timepoints, cognitive improvers showed lower GDF-15 levels than non-improvers (trend). Serum levels of GDF-15 are positively associated with age, possibly because cellular stress and mitochondrial dysfunction events accumulate with age[27]. GDF-15 has a role as mitokine that is induced under conditions of high oxidative stress, mitochondrial DNA damage and protein misfolding. GDF-15 might also control metabolic homeostasis and is implicated in the pathogenesis of metabolic disorders and neurodegenerative processes[27]. Thus, it is plausible that low baseline levels of GDF-15 are associated with proper cognitive function. This is supported by our correlation analyses which revealed a positive correlation between greater reductions of GDF-15 levels and global cognition three years post-surgery (Pearson coefficient = 0.241; p = 0.028, eTable6). The levels of Adiponectin significantly increased from baseline to 3 yrs post-surgery, however, no differences in increased levels were observed between the cognitive improvers and non-improvers. However a greater increase in adiponectin three years post-surgery was associated with improvement of global cognition. Adiponectin has been shown to protect neurons from oxidative stress and to reduce neuroinflammation [28, 29] and other studies report that higher circulating adiponectin levels are associated with better memory and executive function in elderly adults[30]. The post-MBS increase in adiponectin levels in our study may have positive effects on oxidative stress and neuroinflammation improving brain health but is not related to cognitive improvement. Longer follow up period in studies with causal designs are needed to show probably a positive correlation.

Scientific evidence shows that cognitive function improves more significantly and consistently after metabolic (bariatric) surgery than after lifestyle or pharmacological interventions, with notable differences in mechanisms, magnitude, and durability of effects. A systematic review of 31 studies found that bariatric surgery leads to improvements in executive function, memory, attention, and language, with effects observed as early as 6 months and sustained up to at least 48 months[31]. In contrast, non-surgical interventions, after lifestyle or drug treatments (e.g., diet, exercise, GLP-1 agonists), show modest and inconsistent cognitive benefits, often limited to specific domains like attention or processing speed and no significant improvement in executive function or memory[32]. Thereby the sustainability of MBS induced cognitive improvement is at least up to 48 months [31] whereas lifestyle/drugs induced cognitive changes often decline without adherence [32].

Additionally, ApoA1 and MMP-9 were significantly lower, while ANGPT-1 was significantly higher three years post-surgery. ApoA1 can exert anti-oxidant and anti-inflammatory effects[33] and overexpression of ApoA1 may inhibit age related cognitive decline [34]. In our study, ApoA1 levels were significantly decreasing over time compared to baseline levels to three years post-surgery. Moreover, correlation analyses showed that greater reduction of ApoA1 correlated with global cognition post-surgery (Pearson coefficient = 0.326; p = 0.002, eTable 6). This is supported by several studies indicating that lower plasma levels of apolipoprotein A1 (ApoA1) are associated with cognitive decline in specific populations[34]. Further research is needed on this biomarker to unravel its role in cognitive function.

MMP-9 is involved in the breakdown of extracellular matrix and enhanced MMP-9 expression is associated with neuroinflammation and Alzheimer’s disease[35]. The endothelial growth factor ANGPT-1 on the other hand, decreased neuroinflammation and improved cognition in an animal model of vascular dementia[36] and has been shown to help maintain BBB integrity and stabilization of the neurovascular function[37]. In our cohort, lower levels of MMP-9 and higher levels of ANGPT-1 might be partly responsible for improved cognition after MBS, as they might reduce neuroinflammation and improve vascular endothelial function and therewith brain function. This was confirmed by our correlation analyses, where greater increase in ANGPT-1 positively correlated with ability to shift attention (Pearson coefficient = 0.248; p = 0.022, eTable 6). However, no differences in these markers were observed between the cognitive improvers and non-improvers, indicating that underlying mechanisms for cognitive improvement might be more complex.

Six months post-surgery, cognitive improvers showed lower depressive symptoms and lower leptin and CRP levels compared to non-improvers[11]. Three years after surgery, no differences in BDI scores and CRP levels were observed between cognitive improvers and non-improvers. In the case of CRP, this is possibly because levels decreased strongly over time leaving little room for variation at three years. Nonetheless, the profound improvements in BDI scores and CRP levels observed relatively rapidly at six months post-surgery were sustained for three years after surgery in the total cohort. This may suggest that individuals who were non-improvers at the six months’ timepoint may require more time to show similar reductions in depressive symptoms and inflammatory markers than individuals that were cognitive improvers. Consequently, this suggests that rapid mood and/or inflammation improvements after surgery increase the benefit on cognitive performance. In our study many inflammatory markers reduced over time till 3 yrs post-surgery such as SAA, ILb1 and IL6, while adiponectin and BDNF levels increase. However, improvement in these peripheral markers does not necessarily immediately affect neuroinflammation and neuroplasticity, and this processes can remain impaired even after systemic metabolic improvement [38]. Metabolic markers often improve within weeks to months after surgery, but cognitive recovery (e.g., in executive function or memory) may take years, reflecting the time required for neuronal repair, synaptic reorganization, and functional recovery[11, 39]. For example a 6-month pilot trial on time-restricted eating (TRE) in older adults showed metabolic improvements within weeks, such as better glucose homeostasis, but cognitive and cerebrovascular benefits were less immediate and harder to quantify [40]. This suggests a lag of several months between metabolic normalization and observable neurological outcomes. Moreover, cognitive function depends on more than inflammation and hormones. Other required components include: sleep quality improvement, mental health, physical activity, and nutritional adequacy all of which vary post-surgery and impact brain function[41]. It will be of interest to investigate these plasma markers also 5 to 10 years after bariatric surgery and including nutritional status, sleep quality, and physical activity. It will also be of interest to correlate biomarker levels with imaging markers (hippocampal volume, white matter integrity, perfusion, BBB permeability), especially for ANGPT-1. Also causality can be explored using animal models such as knockouts/overexpression of ApoA1 and ANGPT-1, or GDF-15 modulation, to examine effects on synaptic plasticity, neuroinflammation, and cognition.

### Limitations

Our study has some limitations. First, we did not include a control group. To address this limitation, we used alternate versions of cognitive tests to reduce learning effects and applied the 20% change index to identify clinically meaningful improvement. Given the observational design of the BARICO study, this index was the most appropriate method available. However, the absence of a control group limits the ability to establish causality. Future randomized controlled trials are needed to confirm these findings and explore the mechanisms involved. Second, we did not have an equal sex distribution, with only 15% of the cohort being male, although this entirely reflects the general bariatric surgery population, of which 20% is male[42]. However to overcome sex-effects in this study, we also performed sensitivity analyses in women only. This is important as for instance fat distribution and adipocyte characteristics vary between sexes[43]. Therefore future studies should increase the proportion of males in the sample to validate the generalizability of the results. Third, although we controlled for key demographic and clinical variables, we did not include other potential confounders such as preoperative cognitive functioning, sleep quality, or dietary habits. These factors may influence cognitive outcomes and should be considered in future studies with broader scope and larger sample sizes. While we used bivariate correlations to explore associations between cognitive and clinical changes, we acknowledge that multivariate approaches could further disentangle these relationships. However, our focus on biologically plausible and literature-supported associations helps mitigate the risk of spurious findings. Finally, this is an observational study, making it hard to draw causal conclusions. Strengths of the study are: large sample size and the inclusion of variables to better understand potential factors associated with cognitive improvement post-surgery.

## Conclusion

The results of this study indicate that blood pressure and medication use for obesity related comorbidities remained lower three years post-surgery compared to baseline. Additionally, depressive symptoms, systemic inflammation and leptin levels maintained low, while adiponectin and BDNF levels were still higher three years after surgery. Furthermore, 38.6% of the participants showed cognitive improvement with the highest change in ability to shift attention and verbal fluency three years after MBS. Potentially, a more pronounced reduction of leptin and lower GDF-15 might be underlying improved cognition after MBS. Moreover, we suggest that early improvements in systemic inflammation and mood may be more beneficial for cognition post-surgery. Future studies should include a control group and test other mechanisms to clarify cognitive improvement after MBS, including genetics, microbiota, or nutrition. Such studies may provide more insight into the effect of MBS-induced weight loss on cognitive function.

## Supplementary Information

Below is the link to the electronic supplementary material.

Supplementary file1 (DOCX 45 KB)

## Data Availability

No datasets were generated or analysed during the current study.
