# Supplementary material for: Sustained Improvement of Cognition, Mood and Plasma Markers Three Years After Metabolic Bariatric Surgery. The BARICO Study
Source: Obes Surg. 2025 Aug 28;35(9):3888–900. doi: 10.1007/s11695-025-08198-5 (PMC12457502; doi:10.1007/s11695-025-08198-5)
Supplement: Supplementary file 1 — Supplementary file1 (DOCX 45 KB) [file 11695_2025_8198_MOESM1_ESM.docx]

**Supplementary Material**

**eTable 1. Details regarding ELISA assays and electrochemiluminescence.**

| Biomarkers | Company | Catalog number |
| --- | --- | --- |
| Leptin | R&D Systems | DY398 |
| Adiponectin | R&D Systems | DY1065 |
| ApoA1 | Sigma | RAB0610 |
| GDF-15 | R&D Systems | DY957 |
| MMP-9 | R&D Systems | DY911-05 |
| ANGPT-1 | R&D Systems | DY923 |
| SAA | R&D Systems | DY3019 |
| PAI-1 | R&D Systems | DY9387 |
| BDNF | R&D Systems | DY248 |
| TNF-α | Quanterix | 85-0329 |
| IL-1β | Quanterix | 85-0329 |
| IL-6 | Quanterix | 85-0329 |

*Abbreviations: CRP = C-reactive protein, SAA = serum amyloid alpha, TNF-α = tumor necrosis factor alpha, IL = interleukin, PAI-1 = plasminogen activator inhibitor-1, BDNF = brain derived neurotrophic factor, GDF-15 = growth differentiation factor 15, ApoA1 = apolipoprotein A1, MMP-9 = matrix metalloproteinase-9, ANGPT-1 = Angiopoietin 1.*

**eTable 2. Baseline characteristics and cognitive test scores of improvers and non-improvers.**

|  | Improvers (n=39) | Non-improvers (n=62) | p-value |
| --- | --- | --- | --- |
| Baseline characteristics | | | |
| Age, mean ± SD | 45.9 ± 5.03 | 47.39 ± 5.85 | 0.201 |
| Sex, women, n (%) | 37 (94.9) | 48 (77.4) | 0.016 |
| Level of education | | | |
| Low | 2 (5.1) | 2 (3.2) | 0.726 |
| Middle | 21 (53.9) | 38 (61.3) |  |
| High | 16 (41.0) | 22 (35.5) |  |
| Cognition | | | |
| Digit span (sum of forward, backward and sorting) | 28.08 ± 3.46 | 24.33 ± 4.32 | <0.001 |
| Story recall (sum of immediate and delayed recall) | 17.67 ± 5.32 | 16.45 ± 6.71 | 0.341 |
| COWAT | 42.23 ± 8.46 | 35.37 ± 12.02 | 0.002 |
| TAP Flexibility index score | -0.81 ± 6.82 | -5.08 ± 9.37 | 0.015 |
| Compound Z-score | 0.34 ± 0.43 | -0.20 ± 0.73 | <0.001 |

*p-value of differences between improvers and non-improvers based on independent sample t-test or chi-square. Abbreviations: COWAT: controlled oral word association test, TAP = test of attentional performance*

**eTable 3. Missing data per outcome measure before and till 3 years after bariatric surgery.**

| Outcome measure | Number of missings (%) | | | |
| --- | --- | --- | --- | --- |
|  | **Baseline** | **6 months** | **2 years** | **3 years** |
| WC^a^ | 10 (9.3%) | 16 (15.0) | 2 (1.9) | 4 (3.7) |
| Bloodpressure^a^ | 0 (0) | 21 (19.6) | 6 (5.6) | 9 (8.4) |
| Medication use | 0 (0) | 0 (0) | 0 (0) | 1 (0.9) |
| Plasma markers^b^ |  |  |  |  |
| CRP | 14 (13.1) | 2 (1.9) | 4 (3.7) | 4 (3.7) |
| Leptin | 14 (13.1) | 2 (1.9) | 4 (3.7) | 3 (2.8) |
| Adiponectin | 14 (13.1) | 2 (1.9) | 4 (3.7) | 3 (2.8) |
| SAA | 15 (14.0) | 2 (1.9) | 4 (3.7) | 4 (3.7) |
| TNF-α | 14 (13.1) | 2 (1.9) | 4 (3.7) | 3 (2.8) |
| IL-β | 15 (14.0) | 2 (1.9) | 4 (3.7) | 3 (2.8) |
| IL-6 | 14 (13.1) | 2 (1.9) | 4 (3.7) | 3 (2.8) |
| PAI-1 | 14 (13.1) | 2 (1.9) | 4 (3.7) | 4 (3.7) |
| BDNF | 14 (13.1) | 2 (1.9) | 4 (3.7) | 4 (3.7) |
| GDF-15 | 15 (14.0) | 2 (1.9) | 4 (3.7) | 3 (2.8) |
| ApoA1 | 14 (13.1) | 2 (1.9) | 4 (3.7) | 3 (2.8) |
| MMP-9 | 14 (13.1) | 2 (1.9) | 4( 3.7) | 3 (2.8) |
| ANGPT1 | 14 (13.1) | 2 (1.9) | 5 (4.7) | 3 (2.8) |
| TAP flexibility index score | 6 (5.6) | 8 (7.5) | 2 (1.9) | 0 (0) |
| Compound Z-score | 6 (5.6) | 8 (7.5) | 2 (1.9) | 0 (0) |
| BDI^c^ | 3 (2.8) | 3 (2.8) | 5 (4.7) | 9 (8.4) |
| Baecke^c^ | 7 (6.5) | 16 (15.0) | 18 (16.8) | 27 (25.2) |

*Only parameters with missing data are shown in the table. All other parameters did not contain any missing data on one of the timepoints. ^a^ We had a lot of missing data for WC and blood pressure, as during the corona pandemic WC and blood pressure were not standard care anymore due to social distance. ^b^ Not all patients collected blood samples at all timepoints. Many patients did not fill in (the complete) questionnaire and therefore these data were not available or reliable. Abbreviations: WC = waist circumference, CRP = C-reactive protein, SAA = serum amyloid alpha, TNF-α = tumor necrosis factor alpha, IL = interleukin, PAI-1 = plasminogen activator inhibitor-1, BDNF = brain derived neurotrophic factor, GDF-15 = growth differentiation factor 15, ApoA1 = apolipoprotein A1, MMP-9 = matrix metalloproteinase-9, ANGPT-1 = Angiopoietin 1, TAP = test of attentional performance, BDI = beck depression inventory.*

**eTable 4. Plasma concentrations of adipokines and inflammatory markers before and till 3 years after bariatric surgery.**

|  | Median (IQR) | | | | | | |  |  | |
| --- | --- | --- | --- | --- | --- | --- | --- | --- | --- | --- |
| Marker | **Baseline** | **6 months** | | | **2 years** | | **3 years** | **p-value** | |  |
| CRP (µg/ml) | 4.51 (2.96-8.35) | 1.45 (0.58-3.23) | | | 0.77 (0.37-1.48) | | 0.60 (0.30-1.30) | <0.001 | |  |
| Leptin (pg/ml) | 64.6 (50.95 – 85.91) | 13.40 (8.50 – 22.95) | | | 13.40 (8.30 – 21.80) | | 18.95 (11.05 – 33.38) | <0.001 | |  |
| Adiponectin (µg/ml) | 2.20 (1.70 – 2.75) | 2.40 (1.80 – 3.00) | | | 3.60 (2.40 – 5.00) | | 4.80 (3.50 – 7.00) | <0.001 | |  |
| SAA (µg/ml) | 6.94 (4.80 - 15.16) | 2.17 (1.23 - 4.18) | | | 1.96 (1.29 - 3.11) | | 3.70 (2.30 - 6.00) | <0.001 | |  |
| TNF-α (pg/ml) | 3.89 (2.90 – 5.26) | 3.49 (2.78 – 4.22) | | | 3.01 (2.12 – 3.95) | | 5.39 (4.39 – 6.66) | <0.001 | |  |
| IL-1β (pg/ml) | 0.17 (0.07 – 0.37) | 0.06 (0.00 – 0.20) | | | 0.27 (0.17 – 0.44) | | 0.06 (0.04 – 0.07) | <0.001 | |  |
| IL-6 (pg/ml) | 1.76 (1.39 – 3.01) | 1.46 (1.08 – 2.22) | | | 0.50 (0.28 – 0.94) | | 1.22 (0.94 – 1.67) | <0.001 | |  |
| PAI-1 (ng/ml) | 42.25 (29.0-57.2) | | 30.85 (21.6-44.7) | 44.95 (27.3-63.0) | | 46.10 (33.8-63.3) | | <0.001 | |  |
| BDNF (ng/ml) | 15.90 (11.6-19.3) | | 17.6 (13.4-24.0) | 23.6 (18.4-29.1 | | 32.15 (24.7-40.5) | | <0.001 | |  |
| GDF-15, mean ± SD (ng/ml) | 479.02 ± 242.69 | | 515.21 ± 174.52 | 447.70 ± 144.47 | | 468.01 ± 178.08 | | 0.018 | |  |
| ApoA1, mean ± SD (µg/ml) | 535.47 ± 150.94 | | 418.63 ± 113.17 | 342.51 ± 101.19 | | 261.33 ± 112.75 | | <0.001 | |  |
| MMP-9 (ng/ml) | 22.2 (18.2-31.3) | | 21.3 (14.4-28.3) | 15.2 (12.5-19.2) | | 16.8 (13.2-23.1) | | <0.001 | |  |
| ANGPT-1 (ng/ml) | 14.3 (10.4-22.55) | | 18.6 (13.5-23.55) | 36.6 (20.1-48.48) | | 26.15 (21.2-33.9) | | <0.001 | |  |

*Linear mixed model analyses were conducted to examine changes over time. Abbreviations:* *CRP = C-reactive protein, SAA = serum amyloid alpha, TNF-α = tumor necrosis factor alpha, IL = interleukin, PAI-1 = plasminogen activator inhibitor-1, BDNF = brain derived neurotrophic factor, GDF-15 = growth differentiation factor 15, ApoA1 = apolipoprotein A1, MMP-9 = matrix metalloproteinase-9, ANGPT-1 = Angiopoietin 1*

**eTable 5. Differences in anthropometric measures, plasma levels, mood and physical activity after surgery for women only in cognitive improvers and non-improvers.**

|  | Women | | | | | |  |
| --- | --- | --- | --- | --- | --- | --- | --- |
|  | **Improvers (n=37)** | | | **Non-improvers (n=48)** | | |  |
|  | **Baseline** | **3 years** | **p-value** | **Baseline** | **3 years** | **p-value** |  |
| Anthropometric measurements | | | |  |  |  |  |
| TBWL (%), mean± SD | n/a | 32.89 ± 7.98 | n/a | n/a | 31.69 ± 7.96 | n/a |  |
| BMI (kg/m^2^), mean ± SD | 41.9 ± 3.37 | 28.0 ± 4.16 | <0.001 | 41.50 ± 4.78 | 28.29 ± 4.38 | <0.001 |  |
| WC (cm), mean ± SD | 122.26 ± 10.76 | 96.73 ± 13.73 | <0.001 | 122.42 ± 11.0 | 93.66 ± 12.34 | <0.001 |  |
| Blood pressure, mean ± SD |  |  |  |  |  |  |  |
| Systolic (mm HG) | 137.10 ± 16.10 | 122.0 ±23.87 | <0.001 | 136.21 ± 16.0 | 127.93 ± 16.27 | 0.003 |  |
| Diastolic (mm HG) | 84.46 ± 6.83 | 77.71 ± 7.43 | <0.001 | 84.85 ± 8.77 | 78.05 ± 9.38 | <0.001 |  |
| Plasma levels, median (IQR) |  |  |  |  |  |  |  |
| CRP (µg/ml) | 5.57 (3.1-10.6) | 0.60 (0.4-1.5) | <0.001 | 5.74 (3.3-12.3) | 0.70 (0.3-1.3) | <0.001 |  |
| Leptin (pg/ml) | 76.2 (62.90-92.3) | 25.10 (12.4-43.2) | <0.001 | 64.3 (55.0-84.7) | 27.47 (15.0-33.4) | <0.001 |  |
| Adiponectin (µg/ml) | 2.4 (2.0-3.2) | 4.9 (3.7-6.7) | <0.001 | 2.3 (1.8-2.9) | 5.3 (3.9-7.6) | <0.001 |  |
| SAA (µg/ml)^d^ | 8.17 (5.9-18.0) | 4.0 (2.4-8.6) | 0.010 | 12.58 (4.0-14.9) | 4.10 (2.7-6.3) | 0.001 |  |
| TNF-α (pg/ml) | 4.55 (3.0-5.4) | 5.02 (4.1-6.5) | 0.003 | 3.90 (3.1-5.4) | 5.41 (4.7-6.7) | 0.041 |  |
| IL-1β (pg/ml) | 0.19 (0.07-0  .37) | 0.06 (0.05-0.09) | 0.043 | 0.18 (0.08-0.34) | 0.06 (0.04-0.07) | 0.023 |  |
| IL-6 (pg/ml)^b^ | 1.73 (1.4-2.9) | 1.09 (0.8-1.4) | 0.003 | 1.76 (1.4-3.0) | 1.30 (0.9-1.7) | 0.115 |  |
| PAI-1 (ng/ml) | 44.80 (34.8-56.3) | 57.10 (39.5-65.7) | 0.690 | 40.10 (26.7-51.0) | 41.80 (30.1-58.1) | 0.694 |  |
| BDNF (ng/ml) | 17.10 (13.4-24.4) | 34.0 (28.8-41.4) | <0.001 | 15.6 (12.0-19.0) | 32.6 (23.9-39.7) | <0.001 |  |
| GDF-15 (ng/ml), mean ± SD (ng/ml) | 417.76 ± 131.68 | 429.14 ± 129.65 | 0.595 | 517.44 ± 319.21 | 455.95 ± 164.27 | 0.087 |  |
| ApoA1 (µg/ml) | 523.8 (389.7-567.6) | 256.4 (185.0-325.3) | <0.001 | 552.5 (448.8-636.2) | 235.8 (178.1-285.2) | <0.001 |  |
| MMP-9 (ng/ml) | 21.7 (14.6-30.7) | 15.9 (11.3-21.6) | 0.035 | 21.6 (17.5-29.9) | 17.0 (13.4-23.9) | <0.001 |  |
| ANGPT-1 (ng/ml) | 15.4 (10.3-27.7) | 28.0 (22.0-37.2) | 0.001 | 15.2 (11.6-23.8) | 24.6 (20.2-32.7) | <0.001 |  |
| BDI, median (IQR) | 8 (6.0-12.0) | 5.47 (1.0-7.8) | 0.001 | 10.0 (6.0-14.0) | 4.0 (2.0-7.0) | <0.001 |  |
| Baecke, mean ± SD | 7.42 ± 1.25 | 7.90 ± 1.24 | 0.113 | 7.7 ± 1.29 | 8.2 ± 1.3 | 0.037 |  |

*Linear mixed models to asses differences three years after surgery in the cognitive improvers and non-improvers for women only. Abbreviations:* *TBWL = total body weight loss, BMI = body mass index, WC = waist circumference, CRP = C-reactive protein, SAA = serum amyloid alpha, TNF-α = Tumor necrosis factor alpha, IL = interleukin, PAI-1 = plasminogen activator inhibitor-1,* *BDNF = brain derived neurotrophic factor, GDF-15 = growth differentiation factor 15, ApoA1 = apolipoprotein A1, MMP-9 = matrix metalloproteinase-9, ANGPT-1 = Angiopoietin 1, BDI = Beck Depression Inventory.*

**eTable 6. Pearson and spearman correlations between changes in cognitive test scores, changes in obesity indices, plasma markers, BDI and physical activity.**

|  |  | Delta Cognition | | | | |  |
| --- | --- | --- | --- | --- | --- | --- | --- |
|  |  | **Digit Span (sum of Forward, Backward and Sorting)** | **Story Recall (sum of immediate and delayed recall)** | **COWAT** | **TAP Flexibility index score** | **Compound Z-score** |  |
| Delta Anthropometric measurements | | |  |  |  |  |  |
| BMI (kg/m^2^) |  | 0.165 | -0.133 | -0.119 | -0.169 | -0.094 |  |
| WC (cm) |  | 0.002 | -0.141 | -0.033 | -0.049 | -0.118 |  |
| Delta Blood pressure | | |  |  |  |  |  |
| Systolic (mm HG) |  | 0.065 | 0.095 | -0.096 | -0.018 | 0.003 |  |
| Diastolic (mm HG) |  | 0.061 | 0.025 | -0.068 | 0.022 | -0.017 |  |
| Delta Plasma levels | | |  |  |  |  |  |
| CRP (µg/ml) |  | 0.099 | -0.001 | 0.064 | -0.004 | 0.084 |  |
| Leptin (pg/ml) |  | 0.049 | -0.020 | -0.187 | -0.158 | -0.094 |  |
| Adiponectin (µg/ml) |  | -0.006 | -0.148 | -0.106 | -0.186 | -0.226* |  |
| SAA (µg/ml) |  | 0.154 | 0.001 | -0.025 | -0.164 | 0.067 |  |
| TNF-α (pg/ml) |  | 0.207 | -0.100 | 0.133 | -0.081 | 0.026 |  |
| IL-1β (pg/ml) |  | -0.017 | -0.079 | 0.165 | -0.205 | -0.062 |  |
| IL-6 (pg/ml) |  | 0.164 | -0.077 | 0.184 | 0.107 | 0.177 |  |
| PAI-1 (ng/ml) |  | -0.026 | -0.013 | 0.036 | -0.269* | -0.135 |  |
| BDNF (ng/ml) |  | 0.022 | -0.106 | 0.085 | 0.237* | 0.101 |  |
| GDF-15 (ng/ml) |  | 0.085 | 0.159 | -0.006 | 0.157 | 0.241* |  |
| ApoA1 (µg/ml) |  | 0.207 | 0.239* | 0.095 | 0.025 | 0.326** |  |
| MMP-9 (ng/ml) |  | 0.142 | -0.092 | 0.145 | -0.075 | 0.045 |  |
| ANGPT-1 (ng/ml) |  | -0.054 | 0.006 | -0.008 | 0.248* | 0.123 |  |
| Delta BDI |  | 0.216* | 0.169 | -0.205* | -0.161 | 0.020 |  |
| Delta Baecke |  | 0.076 | -0.022 | -0.098 | 0.195 | 0.086 |  |

*Pearson correlation coefficients and spearman correlation coefficients for SAA and IL-1β. Significant correlations are indicated by underscoring these correlation coefficients, p<0.05. Abbreviations: COWAT = Controlled Oral Word Association Test, TAP = Test of Attentional Performance, CRP = C-reactive protein, SAA = serum amyloid A, TNF-α = tumor necrosis factor alpha, IL = interleukin, PAI-1 = plasminogen activator inhibitor 1, BDNF = brain derived neurotrophic factor, GDF-15 = growth differentiation factor 15, ApoA1 = apolipoprotein A1, MMP-9 = matrix metalloproteinase-9, ANGPT-1 = Angiopoietin 1, BDI = Beck Depression Inventory.*

**eTable 7. Pearson and spearman correlations between changes in cognitive test scores, changes in obesity indices, plasma markers, BDI and physical activity for women only.**

|  |  | Delta Cognition | | | | |  |
| --- | --- | --- | --- | --- | --- | --- | --- |
|  |  | **Digit Span (sum of Forward, Backward and Sorting)** | **Story Recall (sum of immediate and delayed recall)** | **COWAT** | **TAP Flexibility index score** | **Compound Z-score** |  |
| Delta Anthropometric measurements | | |  |  |  |  |  |
| BMI (kg/m^2^) |  | 0.183 | -0.173 | -0.096 | -0.188 | -0.105 |  |
| WC (cm) |  | 0.028 | -0.101 | -0.050 | -0.128 | -0.129 |  |
| Delta Blood pressure | | |  |  |  |  |  |
| Systolic (mm HG) |  | 0.035 | 0.072 | -0.123 | 0.031 | -0.027 |  |
| Diastolic (mm HG) |  | 0.013 | 0.037 | -0.125 | 0.032 | -0.073 |  |
| Delta Plasma levels | | |  |  |  |  |  |
| CRP (µg/ml) |  | 0.106 | 0.018 | 0.072 | 0.007 | 0.112 |  |
| Leptin (pg/ml) |  | 0.021 | -0.041 | -0.158 | -0.185 | -0.116 |  |
| Adiponectin (µg/ml) |  | -0.13 | -0.141 | -0.100 | -0.197 | -0.234* |  |
| SAA (µg/ml) |  | 0.142 | 0.020 | -0.029 | -0.201 | -0.055 |  |
| TNF-α (pg/ml) |  | 0.164 | -0.055 | 0.147 | -0.120 | 0.012 |  |
| IL-1β (pg/ml) |  | -0.037 | -0.092 | 0.164 | -0.256* | -0.104 |  |
| IL-6 (pg/ml) |  | 0.162 | -0.129 | 0.198 | 0.068 | 0.137 |  |
| PAI-1 (ng/ml) |  | -0.078 | -0.034 | 0.041 | -0.332** | -0.206 |  |
| BDNF (ng/ml) |  | 0.092 | -0.035 | 0.046 | 0.257* | 0.175 |  |
| GDF-15 (ng/ml) |  | -0.035 | 0.125 | -0.059 | 0.122 | 0.115 |  |
| ApoA1 (µg/ml) |  | 0.195 | 0.259* | 0.062 | -0.028 | 0.305** |  |
| MMP-9 (ng/ml) |  | 0.180 | -0.026 | 0.163 | -0.101 | 0.108 |  |
| ANGPT-1 (ng/ml) |  | 0.017 | 0.052 | -0.057 | 0.278* | 0.190 |  |
| Delta BDI |  | 0.125 | 0.185 | -0.234* | -0.225* | -0.065 |  |
| Delta Baecke |  | 0.071 | -0.048 | -0.086 | 0.205 | 0.082 |  |

*Pearson correlation coefficients and spearman correlation coefficients for SAA and IL-1β. Significant correlations are indicated by underscoring these correlation coefficients, p<0.05. Abbreviations: COWAT = Controlled Oral Word Association Test, TAP = Test of Attentional Performance, CRP = C-reactive protein, SAA = serum amyloid A, TNF-α = tumor necrosis factor alpha, IL = interleukin, PAI-1 = plasminogen activator inhibitor 1, BDNF = brain derived neurotrophic factor, GDF-15 = growth differentiation factor 15, ApoA1 = apolipoprotein A1, MMP-9 = matrix metalloproteinase-9, ANGPT-1 = Angiopoietin 1, BDI = Beck Depression Inventory.*
